# Impact of tracheal cuff shape on microaspiration of gastric contents in intubated critically ill patients: study protocol for a randomized controlled trial

**DOI:** 10.1186/s13063-015-0955-z

**Published:** 2015-09-25

**Authors:** Emmanuelle Jaillette, Guillaume Brunin, Christophe Girault, Farid Zerimech, Arnaud Chiche, Céline Broucqsault-Dedrie, Cyril Fayolle, Franck Minacori, Isabelle Alves, Stephanie Barrailler, Laurent Robriquet, Fabienne Tamion, Emmanuel Delaporte, Damien Thellier, Claire Delcourte, Alain Duhamel, Saad Nseir

**Affiliations:** Critical Care Center, University Hospital of Lille, rue E. Laine, 59037 Lille Cedex, France; Réanimation Polyvalente, CH Dr Duchenne, allée Jacques Monod, BP 609, 62321 Boulogne-Sur-Mer, France; Réanimation Médicale, Hôpital C. Nicolle, 1 rue de Germont, 76031 Rouen Cedex, France; Laboratoire de Biochimie et Biologie Moléculaire, Université de Lille et Pôle de Biologie Pathologie Génétique du CHRU de Lille, 59000 Lille, France; Réanimation Médicale et Infectieuse, CH de Tourcoing, 115 rue du Président Coty, 59208 Tourcoing Cedex, France; Réanimation Polyvalente, Hôpital Victor Provo, 17 bd Lacordaire, BP 359, 59056 Roubaix, France; Service de réanimation polyvalente, 130 Avenue Louis Herbeaux BP 6367, 59140 Dunkerque, France; Réanimation Polyvalente, CH Saint Philibert, 115 Rue du Grand But, BP 249, 59462 Lomme Cedex, France; Réanimation Médicale, CH de Valenciennes, Avenue Desandrouin, BP479, 59322 Valenciennes Cedex, France; Réanimation Polyvalente, CH Dr Schaffner, 99 route de La Bassée, BP8, 62307 Lens Cedex, France; Clinique de Santé Publique, plateforme d’aide méthodologique, 59037 Lille Cedex, France; Medical School, Lille University, 1 place de Verdun, 59000 Lille, France

## Abstract

**Background:**

Ventilator-associated pneumonia (VAP) is the most common infection in intubated critically ill patients. Microaspiration of the contaminated gastric and oropharyngeal secretions is the main mechanism involved in the pathophysiology of VAP. Tracheal cuff plays an important role in stopping the progression of contaminated secretions into the lower respiratory tract. Previous in vitro studies suggested that conical cuff shape might be helpful in improving tracheal sealing. However, clinical studies found conflicting results. The aim of this study is to determine the impact of conical tracheal cuff shape on the microaspiration of gastric contents in critically ill patients.

**Methods/Design:**

This prospective cluster randomized controlled crossover open-label trial is currently being conducted in ten French intensive care units (ICUs). Patients are allocated to intubation with a polyvinyl chloride (PVC) standard (barrel)-shaped or a PVC conical-shaped tracheal tube. The primary objective is to determine the impact of the conical shaped tracheal cuff on abundant microaspiration of gastric contents. Secondary outcomes include the incidence of microaspiration of oropharyngeal secretions, tracheobronchial colonization, VAP and ventilator-associated events. Abundant microaspiration is defined as the presence of pepsin at significant level (>200 ng/ml) in at least 30 % of the tracheal aspirates. Pepsin and amylase are quantitatively measured in all tracheal aspirates during the 48 h following inclusion. Quantitative tracheal aspirate culture is performed at inclusion and twice weekly. We plan to recruit 312 patients in the participating ICUs.

**Discussion:**

BEST Cuff is the first randomized controlled study evaluating the impact of PVC tracheal-cuff shape on gastric microaspirations in patients receiving invasive mechanical ventilation. Enrollment began in June 2014 and is expected to end in October 2015.

**Trial registration:**

ClinicalTrials.gov Identifier: NCT01948635 (registered 31 August 2013).

## Background

### Ventilator-associated pneumonia as a common intensive care unit-acquired infection

Ventilator-associated pneumonia (VAP) is the most common ICU-acquired infection in intubated critically ill patients [1, 2]. VAP is associated with prolonged duration of mechanical ventilation, and ICU stay, increased antibiotic use, mortality and additional cost [3–6]. During the last decades, VAP prevention has become a major quality-indicator in ICU patients [7, 8].

### Microaspiration and pathogenesis of ventilator-associated pneumonia

Microaspiration of gastric and oropharyngeal contaminated secretions represents the primary mechanism of VAP pathogenesis [9–11]. Whereas microaspiration of contaminated secretions is common in intubated critically ill patients, many of them do not develop subsequent VAP. Local and general defense mechanisms frequently prevent the progression from tracheobronchial colonization to ventilator-associated tracheobronchitis, and VAP. However, when these mechanisms are insufficient and/or when quantity and/or virulence of aspirated microorganisms are high, lower respiratory tract infections develop in critically ill patients [9].

Many risk factors for microaspiration have been identified and could be classified into those related to a tracheal tube, to mechanical ventilation, to enteral nutrition, and to the patient. Impossible closure of the vocal cords, longitudinal folds in the PVC-cuffed tracheal tube, and underinflation of the tracheal cuff under 20 cmH_2_O are the main factors related to the tracheal tube. Zero positive end-expiratory pressure, low peak inspiratory pressure, and tracheal suctioning are those related to mechanical ventilation. Enteral feeding and nasogastric tubes are also important risk factors for microaspiration because they are associated with gastroesophageal reflux, gastric distension, and loss of the anatomic integrity of the lower esophageal sphincter. Patient-related risk factors are supine position, sedation and paralytic agents use, hyperglycemia, viscosity of the oropharyngeal secretions above the cuff, tracheal diameter, and tracheal tube mobilization [9].

Pepsin derives from pepsinogen and is released from the chief cells in the stomach [12]. Many animal and human studies have suggested that its presence in tracheal secretions reflects gastric microaspiration in intubated subjects [13–16]. Using pepsin as a marker of gastric microaspiration in intubated critically ill patients is easy to perform routinely. Benefits in using this quantitative biomarker are that it is a noninvasive procedure and that it offers the possibility to quantify microaspiration. Using a quantitative marker to diagnose microaspiration is important because VAP occurrence is closely correlated to the amount of aspirated microorganisms [17, 18].

Evaluating the impact of preventive measures on microaspiration of gastric contents is an interesting strategy because microaspiration is more common than VAP. Therefore, before performing large studies aiming to determine the impact of a preventive measure on VAP incidence, a study evaluating the impact of such a measure on the incidence of microaspiration would probably be helpful to confirm its efficiency.

### Conical cuff shape and ventilator-associated pneumonia

Tracheal cuff shape seems to play an important role in preventing microaspiration and VAP. A conical (tapered)-shaped tracheal cuff insures a permanent sealing zone between the external cuff diameter and the internal trachea diameter, whatever the size and the diameter of the trachea. This permanent contact between the cuff and the trachea would prevent longitudinal folds and reduce microaspiration [19].

Three in vitro studies have examined the relation between conical cuff shape and fluid or air leakage [20–22]. Dave et al. demonstrated that there was no significant difference between a polyurethane conical versus a cylindrical cuff on fluid leakage around the cuff when tracheal diameter is small [20]. In contrast, conical shape was more effective than cylindrical shape for reducing fluid leakage in large-diameter tracheas. In another study, Zanella et al. did not find any significant difference in fluid leakage between polyurethane conical versus cylindrical cuffs [22]. Madjpour et al. reported that conical-shaped PVC cuffs improve air-sealing, compared with the PVC cylindrical cuffs [21]. However, no significant difference was found between conical versus cylindrical polyurethane cuffs. A recent animal study evaluated the impact of PVC cuff shape on fluid leakage, using methylene blue as a marker for leakage. The authors concluded that leakage was significantly reduced in animals intubated with conical-cuffed tubes, compared with those intubated with cylindrical-cuffed tubes [23].

D’Haese et al. performed a randomized controlled study to evaluate the impact of conical cuff shape, compared with barrel cuff shape, on microaspiration of blue dye in 60 patients scheduled for lumbar surgery [24]. They reported that conical cuff shape was associated with reduced leakage of dye diagnosed using fiberoptic bronchoscopy. A recent multicenter randomized controlled study evaluated the impact of polyurethane and/or conical cuffs on tracheal colonization [25]. A total of 621 patients with an expected duration of mechanical ventilation longer than 2 days were included with cuffs composed of cylindrical PVC (n = 148), cylindrical polyurethane (n = 143), conical PVC (n = 150), or conical polyurethane (n = 162). No significant difference was found regarding the incidence of tracheal colonization or VAP between the different study groups. However, tracheobronchial colonization is probably not a good marker for microaspiration because antibiotic treatment might influence the rate of colonization. Whereas tracheobronchial colonization mainly results from aspiration of endogenous bacteria, contamination by exogenous bacteria is still possible [26, 27]. Several studies have shown that exogenous contamination could result from contaminated aerosols, tracheal suctioning or flexible bronchoscopy. Therefore, no definite conclusion could be drawn based on the results of the TOP-cuff trial.

### Study aim

The aim of this study is to determine the impact of a PVC conical-cuffed tracheal tube, compared with a PVC standard (barrel)-cuffed tracheal tube on abundant microaspiration of gastric contents in patients with a predicted duration of mechanical ventilation of at least 48 h. Secondary objectives include the impact of a conical-shaped tracheal cuff on microaspiration of oropharyngeal secretions, tracheobronchial colonization, VAP, and ventilator-associated event (VAE) incidence.

## Methods/Design

### Design

**Fig. 1:**
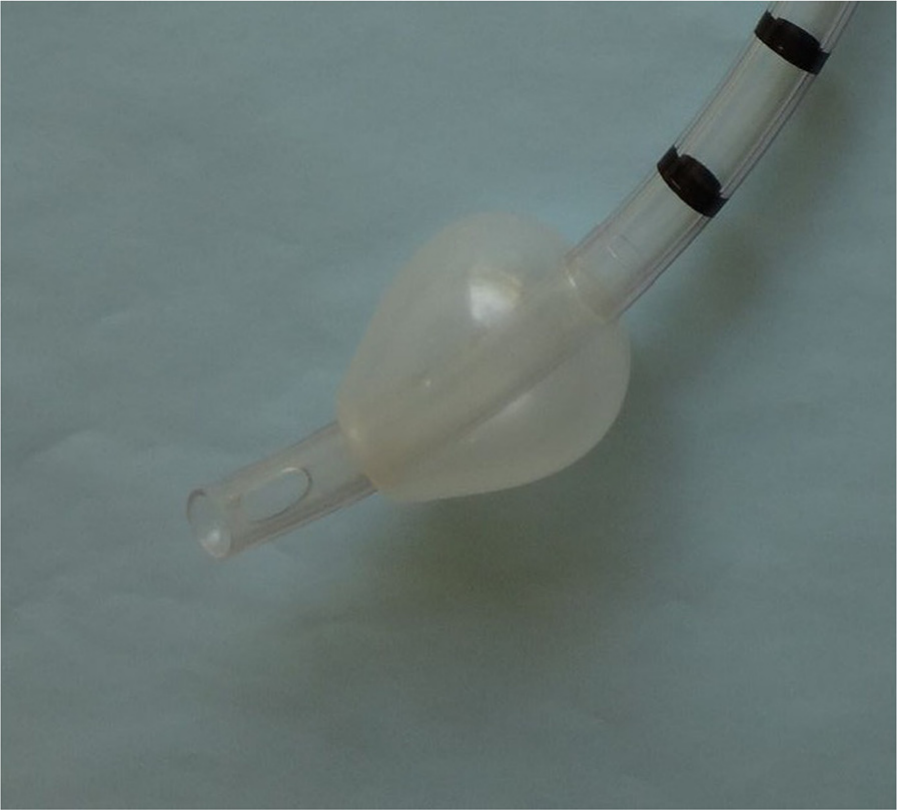
Conical tracheal cuff

**Fig. 2:**
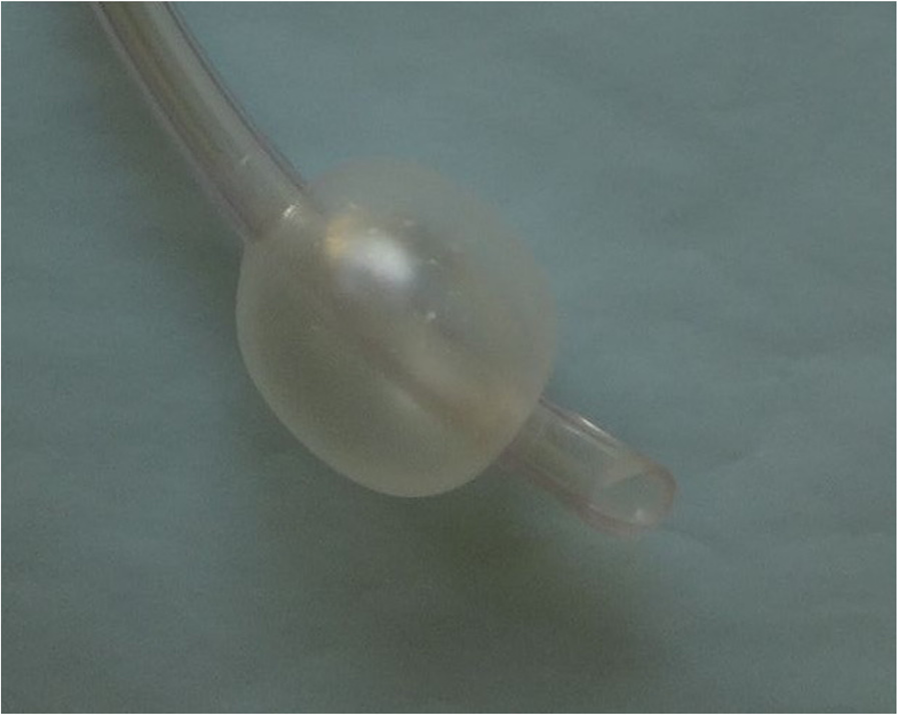
Standard tracheal cuff

BEST CUFF is a multicenter, cluster randomized crossover controlled and open-label trial performed in patients receiving mechanical ventilation in the ICU. Because tracheal intubation is an urgent procedure in ICU patients, the randomization was performed on the participating ICUs, and not on the patients. These ICUs were randomized into two balanced groups, according to an intervention sequence. Using this design, half of ICUs are randomized to use a PVC conical cuff (Fig. 1) in the first 1-month period and a PVC standard cuff (Fig. 2) in the second 1-month period. The other half of the ICUs will use the two interventions in the reverse sequence.

**Fig. 3:**
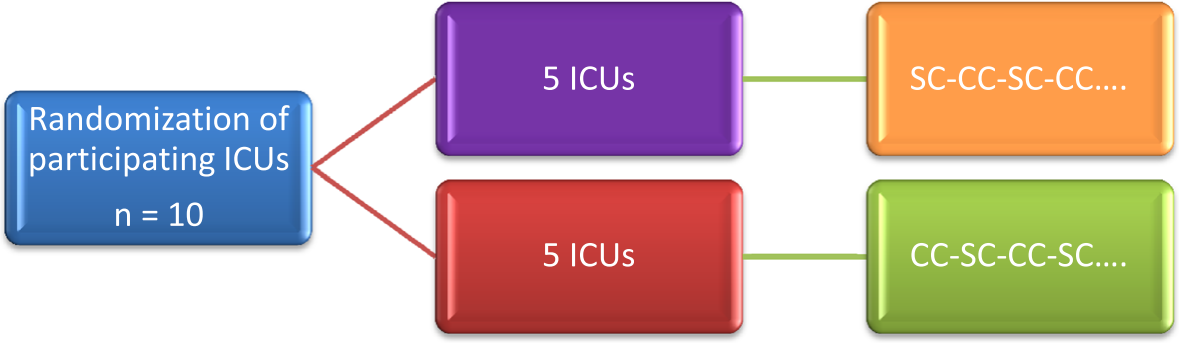
Randomization in participating intensive care units (ICUs). CC, conical cuff; SC, standard cuff

Each ICU recruits distinct subjects during the repeated 2-month periods using the assigned intervention sequence, meaning that each intervention sequence started must be completed (Fig. 3).

### Ethical aspects

The study protocol and patients (or next of kin) information documents were approved by the Ethical Committee, and Institutional Review Board of the Lille University Hospital (Comité de Protection des Personnes Nord-Ouest, approved 2 July 2013, registration 2013 A00534 41).

Complete and fair information is provided to the patient or to his representative. An informed written consent is obtained before enrollment. When consent is given by proxies, the patient is informed as soon as possible, and his written consent is obtained.

### Participating units

This study is currently conducted in ten French ICUs, including three located in university hospitals and seven in general hospitals.

### Study population

Inclusion criteria are age ≥18 years, intubation in the ICU with the study tracheal tube, an expected duration of mechanical ventilation of at least 48 h after inclusion. Exclusion criteria are age <18 years, pregnancy, patient or proxy refusal, contraindication for enteral feeding, intubation for >72 h at screening for eligibility in the trial, tracheostomy at ICU admission, previous enrollment in this study, or the inclusion in another study that may interfere with this trial.

### Randomization

The randomization procedure (1:1 assignment intervention sequence) was determined using a computer random-number generator and was conducted by the Statistics Department at Lille University Hospital (Lille, France) by a person not associated with the study. The two interventions studied are intubation using PVC conical-cuffed tracheal tubes (TaperGuardR, Covidien, Astlone, Ireland) and intubation using PVC standard (barrel)-cuffed tracheal tubes (Hi-LoR, Covidien, Astlone, Ireland). When reintubation is required, a tracheal tube with the same cuff shape as the one used for the first intubation is used.

### Definitions

Abundant microaspiration is defined by significant pepsin level (>200 ng/ml) in >30 % of tracheal aspirates during the 48 h following inclusion. This cut-off was calculated based on the results of previous studies [13, 14].

Microaspiration of oropharyngeal secretions is defined by the presence of alpha-amylase at significant level (1800 UI/l) [28, 29] in tracheal aspirates. Tracheobronchial colonization is defined by positive endotracheal sample without clinical and radiological signs of VAP.

VAP is defined as the presence of radiological and clinical signs consisting of a new and persistent infiltrate on the chest radiograph associated with two of the three following criteria: purulent tracheal aspirates, hyperthermia >38 °C or hypothermia <36 °C and peripheral leucocytosis >10,000/μl or <1,500/μl. A microbiological confirmation is required using tracheal aspirate ≥10^5^ CFU/ml or bronchoalveolar lavage ≥10^4^ CFU/ml [30]. Patients with only clinical and radiological signs without microbiological confirmation are considered to have a suspected VAP. Other ICU-acquired infections are defined elsewhere [31].

Ventilator-associated events (VAE) are defined as a sustained increase in ventilator support (minimum PEP increase >2.5 cm H_2_O, or minimum FiO_2_ increase >15 %) after >2 days of stable or decrease settings [32]. Patients with PEP >75 cm H_2_O or FiO_2_ > 70 % during the first 3 days of mechanical ventilation are tested for these VAC only if a higher stabilization is observed (PEP <5 cm H_2_O, FiO_2_ < 40 % during >2 days).

### Study protocol

**Fig. 4:**
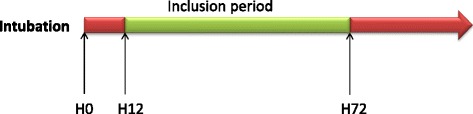
Timing of tracheal aspirate collection for pepsin and alpha-amylase measurements

Inclusions are performed 24 h/24 h, every weekday. After inclusion, tracheal aspirates are collected for 48 h to measure pepsin and amylase. When informed consent is obtained <12 h after intubation, tracheal aspirates collection is started at least 12 h after intubation (Fig. 4). All tracheal aspirates are stored at -20 °C in each hospital’s laboratory, and subsequently sent to the central laboratory at the Lille University Hospital, where all measurements of pepsin and amylase are blindly performed. Quantitative measurement of pepsin is performed by an ELISA technique, and salivary amylase activity is calculated by the difference between total and pancreatic amylase activity. The volume required to perform this analysis is 1 to 2 ml in each tracheal aspirate.

To diagnose tracheobronchial colonization, quantitative tracheal aspirate is performed after intubation, and two times a week. In patients with suspected VAP, quantitative tracheal aspirate or bronchoalveolar lavage is performed to confirm the diagnosis. The choice between tracheal aspirate culture or bronchoalveolar lavage is left to the investigator’s discretion, based on routine practice in each ICU. Investigators prospectively follow patients to detect clinical, radiological or microbiological signs of suspected VAP or VAE. Patients will be followed until ICU discharge or day 28, whichever happens first.

### Care standardization

Cuff pressure is checked with a manual manometer every 8 h and kept around 25 cm H_2_O. Patients are ventilated in a semi-recumbent position. Female and male patients are intubated with tracheal tubes sized 7.5 and 8 mm; respectively. Elevation of the head of the bed is checked every 3 h. Oropharyngeal decontamination is performed with 0.10 % chlorhexidine every 3 h. Mechanical ventilation is performed with a positive end-expiratory pressure of at least 5 cm H_2_O, except when contraindicated. Weaning for mechanical ventilation is checked every day to reduce the duration of tracheal intubation. Systematic stress ulcer prophylaxis is not routinely recommended. The shortest duration of sedation is recommended, using a nurse-driven protocol. Tracheal suctioning is performed using an open system. Subglottic secretion drainage is not used, and the respiratory circuit is not routinely changed in study patients.

### Data collections

At the time of inclusion, the following baseline characteristics are recorded: age, sex, height, body weight, date of hospital admission, date of ICU admission, patient location before ICU admission (home, hospital for >24 h), date of intubation and mechanical ventilation, preexisting comorbidities (diabetes and COPD, chronic restrictive respiratory failure, chronic heart failure, cirrhosis Child B or C, chronic renal failure, esogastroduodenal reflux, immunosuppression), McCabe score, Simplified Acute Physiology Score II, SOFA score, and reason for the ICU admission.

At the time of randomization, the following characteristics are recorded: SOFA score, duration of enteral feeding before randomization, nasal or oral position of the tracheal tube, cuff shape, and tube size.

During the 48 h following randomization, the variables listed below are recorded: ventilatory mode, mean PEEP, mean airway pressure, mean peak pressure, mean cuff pressure, highest FiO_2_, nitric monoxide use, aerosols, vasopressor support, enteral feeding volume, gastric residual volume, stress ulcer prophylaxis, use of prokinetic agents, vomiting, sedation, neuromuscular blockers, Glasgow Coma Score, mean head of bed elevation, prone position, patient mobilization (on chair), patient mobilization outside ICU room, accidental extubation, reintubation, and death.

Until day 28 or until ICU discharge, the following variables are being collected: sedation, transfusion, tracheotomy, extubation failure, accidental extubation, reintubation, shock, transport outside the ICU, mean head of bed elevation, use of proton pump inhibitors, ICU-acquired infection other than VAP (bacteremia, ventilator-associated tracheobronchitis, intravascular catheter-related infection, urinary tract infection, soft tissue infection, and others), duration of antibiotic treatment, duration of mechanical ventilation, length of ICU stay, death, data related to VAE, pepsin and alpha-amylase levels, VAP, and tracheobronchial colonization (Table 1).

**Table 1:** Study flowchart follow-up

	Inclusion	H12 to H72	Until Day 28 or ICU discharge	Suspected VAP
Eligibility : check inclusion and exclusion criteria	X			
Randomization	X			
Quantitative TA culture	X		two times a week	X
Pepsin and amylase measurement in all TA for 48 h		X		
Clinical and radiological signs of VAP			X	
Quantitative TA culture or BAL				X
Clinical data			X	

*ICU* intensive care unit; *TA* tracheal aspirate; *VAP* ventilator-associated pneumonia; *BAL* bronchoalveolar lavage

### Blinding

Blinding of physicians, nurses and patients is not feasible in this study. However, pepsin and amylase measurements are performed blindly. VAP diagnosis will be confirmed by at least two independent blinded physicians.

### Trial conduct and data monitoring

Before the start of patient enrollment, all physicians and health-care workers received briefing on the study protocol and data collection in the electronic case report form (eCRF). All documents required for this study are available in each ICU in a folder dedicated to the study. Physicians and/or a clinical research assistant are in charge of daily patient screening and inclusion. The principal investigator is available to all healthcare workers to answer all questions regarding the proper conduct of the study.

### Study outcomes

#### Primary endpoint

The primary endpoint is the proportion of patients with abundant microaspiration of gastric contents. This endpoint will be measured during the 48 h following inclusion in the study.

#### Secondary endpoints

The secondary endpoints include the following:

1. Proportion of tracheal aspirates positive for alpha-amylase per patient
2. Proportion of patients with tracheobronchial colonization, and bacterial concentration per patient
3. Proportion of patients with VAP
4. Proportion of patients with VAE
5. Proportion of patients with at least one ICU-acquired infection
6. Antimicrobial-free days
7. Invasive mechanical ventilation-free days
8. Length of ICU stay
9. Proportion of patients who die in the ICU

The proportion of tracheal aspirates positive for alpha-amylase per patient will be measured during the 48 h following inclusion in the study. Other secondary endpoints will be measured from inclusion through day 28 or to final extubation if it happens before day 28.

### Sample size

The main objective of this study is to demonstrate, in intubated critically ill patients, the superiority of conical cuff shape in reducing abundant microaspiration of gastric contents, compared with standard cuff shape (control group). Based on previous studies [13, 14], the expected incidence of abundant microaspiration of gastric contents is 50 % in control group. We assumed that the use of conical-cuffed tracheal tubes could reduce this incidence to 30 % (that is, an absolute and relative risk reduction of 20 % and 40 %, respectively). To detect this effect size, with a two-sided alpha risk of 5 %, and a power of 80 %, 90 patients would be required per group in a conventional parallel*-*group randomized trial. Since the experimental design is a cluster randomized crossover*,* we adjusted the study sample size to take into account the correlation between outcomes of patients within ICU (namely intraclass correlation coefficient, ICC) [33]. We did not take into account the inter-period correlation (correlation between different patients included within the same ICU but at different periods), in order to maximize the statistical power. Using the formula commonly used to plan a randomized cluster trial by considering each ICU as a pool of two independent clusters (one within each arm), assuming an ICC of 0.035 (corresponding to the upper quartile of ICC values from 31 primary care researches) [34], an expected total number of patients included per ICU of 30 patients, we calculated that 140 patients would be required per group. To account for an anticipated rate of 10 % of patients without any tracheal secretions, we planned to recruit a total of 312 patients.

### Statistical analysis

**Fig. 5:**
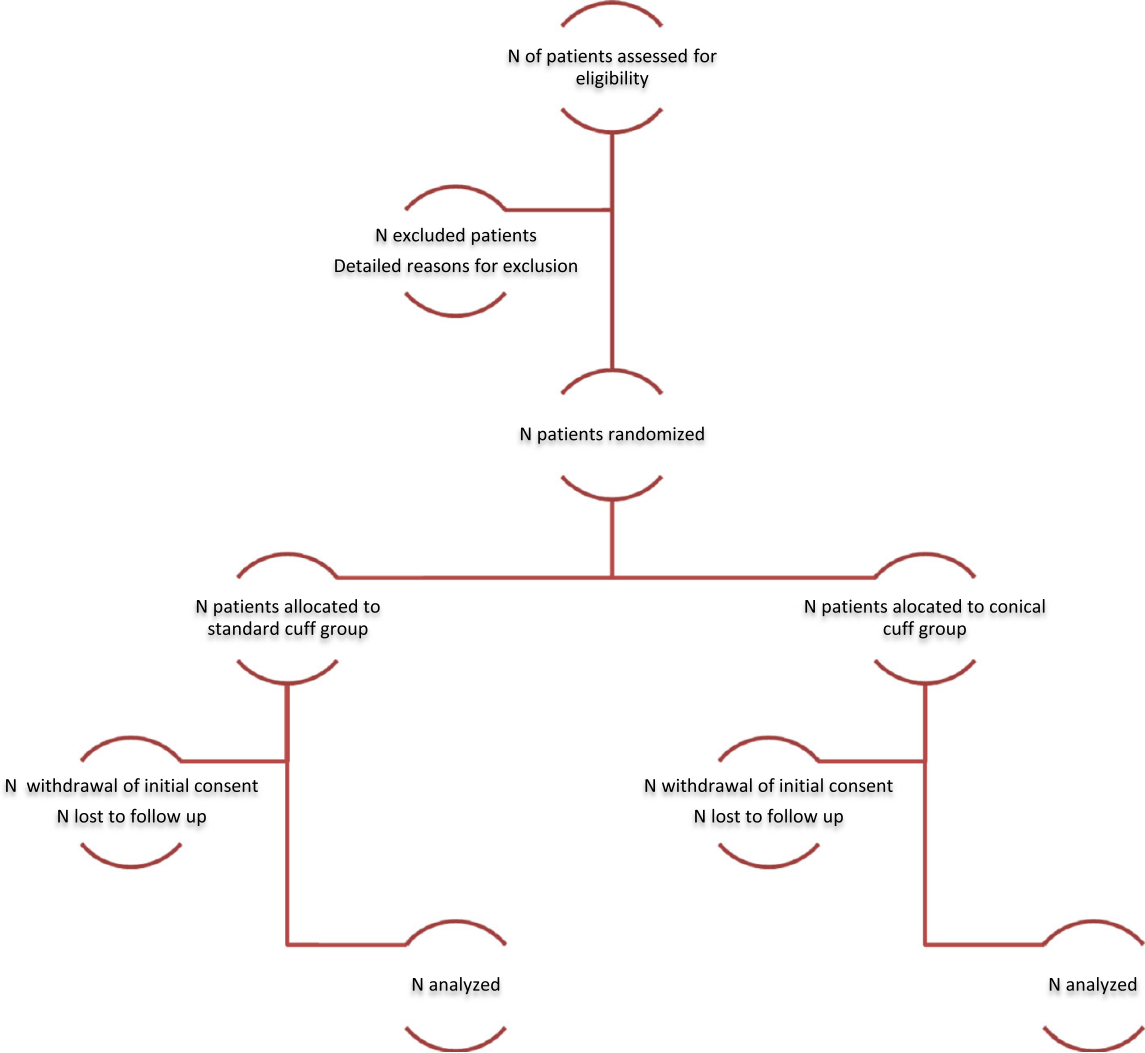
Flow diagram of Best cuff trial according to CONSORT

Statistical analyses will be independently performed by the Biostatistics Department of University of Lille. Data will be analyzed using the SAS software (SAS Institute Inc, Cary, NC, USA), and all statistical tests will be performed with a two-tailed alpha risk of 0.05. Statistical analysis will be performed on an intention-to-treat basis. A detailed statistical analysis plan will be written and finalized prior to the database lock. The final report will be written, based on the CONSORT statement recommendations (Fig. 5).

Baseline characteristics will be compared between the two study groups. Quantitative variables will be expressed as mean (standard deviation), and median (interquartile range). Qualitative variables will be expressed as frequencies and percentages. Normality of distribution will be assessed graphically and using the Shapiro-Wilk test. Chi-square (or Fischer’s exact test), and Student *t*-test (or Mann Whitney test) will be performed to compare qualitative, and quantitative variables, as appropriate.

#### Primary endpoint

The proportion of patients with abundant microaspiration will be compared between the two group using a generalized linear mixed model including group as fixed effect, and center and center*time period as the random effects. Missing data for the primary endpoint are being handled by multiple imputation using chained equations (m = 10 imputations using primary endpoint and patient’s characteristics at inclusion).

#### Secondary endpoints

A generalized linear mixed model including group as the fixed effect and center and center*time period as random effects will also be used to compare each secondary endpoints between the two groups. For quantitative endpoints, the normality of the model residuals will be checked. In cases of non-normal distribution of residuals (except if a log-transformation could be applied to normalize the data), quantitative endpoints will be compared using a Mann-Whitney *U* test.

## Discussion

Whether the conical cuff shape of the tracheal tube is efficient in reducing microaspiration of contaminated oropharyngeal and gastric secretions is unknown. Previous clinical studies reported conflicting results [24, 25] and had several limitations, precluding any definite conclusion. Our study is sufficiently powered to detect a significant difference in microaspiration of gastric contents between patients intubated with conical-cuffed tracheal tube and those intubated with standard-cuffed tracheal tubes. One of the strengths of this study is the use of quantitative measurement of pepsin as a marker of microaspiration. Pepsin is probably the most accurate, currently available, marker for microaspiration [9]. Whereas technetium-99 is the gold standard for the diagnosis of microaspiration of gastric contents [35], its use is not allowed outside the Radiology Department. The transport of critically ill patients outside the ICU has a considerable potential for misadventure and could be a life-threatening endeavor [36]. In addition, transport outside the ICU was identified as a risk factor for VAP [37]. Other markers for microaspiration, such as blue dye, and tracheobronchial colonization have several limitations, such as the qualitative measurement of microaspiration and the presence of confounding factors [9]. Alpha-amylase is a new marker for microaspiration of oropharyngeal secretions. Two recent studies [28, 38] reported promising results for its use in patients with aspiration pneumonia and in small cohorts of critically ill patients. However, our group found a moderate accuracy of alpha-amylase in diagnosing microaspiration compared with pepsin [29]. Further large clinical studies should validate its accuracy in patients with VAP.

We exclude patients with contraindication for enteral nutrition and those with gastrostomy because these factors might influence the primary outcome. We aimed to include patients early during invasive mechanical ventilation in order to increase the probability of them remaining under invasive mechanical ventilation and to have a complete period of 48 h during which tracheal aspirates are collected for pepsin and alpha-amylase measurements.

Nevertheless, we start collection of tracheal aspirates for pepsin measurements at least 12 h after intubation because this procedure is a well-known risk factor for microaspiration. As pepsin half-life is relatively short (3 to 4 h), this would probably allow a better interpretation of pepsin measurement results.

Subglottic secretion drainage is an effective measure in preventing VAP [39, 40]. However, this measure was not clearly recommended when our trial was designed, and its use was not generalized to all ICUs. In addition, one could argue that it is probably more accurate to evaluate the efficiency of these preventive measures, that is, the subglottic secretion drainage and conical shape, separately.

If the study hypothesis is confirmed, further studies should confirm the impact of conical cuff shape on the incidence of VAP.

## Trial status

Enrollment is ongoing. Inclusion started in June 2014. By March 2015, 201 patients had been included. Recruitment is expected to be complete in October 2015.

## Abbreviations

PVC: Polyvinyl chloride
VAE: Ventilator-associated event
VAP: Ventilator-associated pneumonia
